# Illness perceptions in Raynaud’s phenomenon: A qualitative study

**DOI:** 10.1177/20551029251355350

**Published:** 2025-07-17

**Authors:** Mashal Hajar Safi, Paula Smith, Johanna Meyer, Jo Daniels

**Affiliations:** 1 1555 University of Bath, UK; 2 1982 North Bristol NHS Trust, UK

**Keywords:** illness perceptions, Raynaud’s phenomenon, qualitative, common-sense model (Leventhal)

## Abstract

Raynaud’s Phenomenon is a condition characterised by vasoconstriction of the extremities and can have a detrimental impact on people’s lives. The treatment options for patients currently offered through healthcare services, e.g., in the United Kingdom (UK), are insufficient. This study provides a theoretical basis for future interventions by presenting insights into experiences of living with Raynaud’s. Using an inductive-deductive approach, our thematic analysis of 19 semi-structured interviews with people living with Raynaud’s in the UK was informed by the common-sense model of self-regulation. Three interrelated themes were generated to capture the illness experiences of people with Raynaud’s: 1) Illness representations, 2) Coping response, and 3) Advice and guidance. Together, these themes provide insight into illness perceptions, coping styles and factors that may contribute to adherence to self-management. The findings suggest that a stepped care approach to managing Raynaud’s may be beneficial.

## Introduction

Raynaud’s Phenomenon (RP) is a condition affecting 3–21% of the global population (Carpentier et al., 2006) and is characterised by vasoconstriction of the extremities, causing distress (Shapiro and Wigley, 2017), moderate to severe pain, numbness and tingling, often resulting in temporary paralysis (Temprano, 2016). Primary RP accounts for 80–90% of cases (National Institute for Health and Care Excellence [NICE], 2020). This type refers to the presence of RP symptoms with no known cause or underlying pathology (Pauling et al., 2019). In secondary RP, the symptoms are attributable to underlying conditions such as Systematic Sclerosis (Pauling et al., 2019). Approximately 14–37% of primary cases progress onto the more severe secondary RP (Maundrell and Proudman, 2014). RP is more prevalent in colder than in warmer climates (Maricq et al., 1997). In the United Kingdom (UK), first-line treatment of RP is self-management through lifestyle and behavioural changes (NICE, 2020). Recommended changes include avoiding cold exposure, keeping the whole body warm as well as using local warming for hands and feet, stopping smoking and reducing caffeine consumption, managing stress, and avoiding triggers. These recommendations are based on guidelines from the European Society for Vascular Medicine (Belch et al., 2017), guidelines from the British Society for Rheumatology (Denton et al., 2024), and expert opinion based on review of available evidence (e.g., Herrick, 2017; Nawaz et al., 2022; Wigley and Flavahan, 2016). However, a recent systematic review highlighted that there are currently no effective behaviour-change interventions or materials to help support patients to establish and maintain changes recommended by NICE (Daniels et al., 2018).

Cold exposure and temperature changes are the main triggers of RP, however up to 30% of episodes are associated with emotional stress and anxiety (Brown et al., 2001; Pauling et al., 2018) with further research indicating the signifcance of anxiety in symptom severity (Irving and Daniels, 2024). While research has identified effective treatment interventions for stress-reduction and anxiety (Olatunji et al., 2010) in other health conditions (Niazi and Niazi, 2011), there are currently no equivalent non-pharmacological interventions recommended for RP (Daniels et al., 2018; NICE, 2020). NICE-recommended second-line treatment for RP consists of pharmacological interventions such as calcium channel blockers (CCB). Compliance to these interventions is low, treatments are effective in less than 50% of cases, and 75% of those who do adhere experience adverse side effects such as dizziness, tachycardia, and a substantial reduction in quality of life (NICE, 2020; Pauling et al., 2018). Treatment efficacy of current pharmacological treatments for RP is below a clinically meaningful difference and individual factors such as age and episode frequency as well as severity influence pharmacological treatment outcomes (Khouri et al., 2019).

Recent studies have emphasised a need for the development of non-pharmacological interventions for RP (Daniels et al., 2018; Hughes et al., 2015; Khouri et al., 2019). In their systematic review of behaviour change interventions for RP, Daniels et al. (2018) found that seven out of eight studies on this topic relied on biofeedback as their behaviour change treatment and that no conclusions could be reached about interventions’ efficacy and safety. Given that episodes of RP can be triggered by factors amenable to self-management (cold and stress), a concerted research effort is needed to design and test behaviour change interventions that aid self-management (Daniels et al., 2018). To develop effective behaviour change interventions, the perspectives and experiences of those who will be using the intervention need to be understood (Araújo-Soares et al., 2019). This is the aim of the current study. The only qualitative research to date exploring the experiences of RP used focus groups (Pauling et al., 2018). Yet this study focused only on the rare secondary RP in Systemic Sclerosis, which is qualitatively different from primary RP (Daniels et al., 2018; Mayes, 2003) and experienced alongside significant other debilitating symptoms. There are no known studies that explore the individual experience of RP, despite the need to better understand those with the illness, and the importance of qualitative research as preliminary evidence for developing behaviour change interventions (Akard et al., 2013; Medical Research Council, 2019).

Extensive research demonstrates the influence of illness representations on health outcomes, such as adherence to treatment (Lamiani et al., 2015), quality of life, and physical functioning (Anghel et al., 2019; Brijs et al., 2019; Petrie et al., 1996). An illness representation is an individual’s understanding of a given health threat, including how it may be managed (Leventhal et al., 2016). A frequently used (see e.g., Hagger et al., 2017; Humphreys et al., 2021; Ní Chéileachair et al., 2022; O’Cathain et al., 2020) model for understanding illness experiences of long-term health conditions is Leventhal’s common-sense model of self-regulation (CSM; Leventhal et al., 1980), a widely accepted framework that is instrumental in understanding illness self-management (McAndrew et al., 2008), and the dynamic and multidimensional processes that underly health and coping (Leventhal et al., 2016; Richardson et al., 2017). The CSM describes individuals’ illness representations along five distinct dimensions (Leventhal et al., 2016): (1) *identity*, referring to the label or name given to the health threat and the symptoms that are perceived to correspond with the illness; (2) *cause,* referring to the individual’s perceived cause and manifestation of the illness and symptoms; (3) *timeline,* referring to the perceived duration of the illness; (4) *consequence,* which includes the effects of the illness and the expected physical, social and psychological outcomes, and (5) *controllability,* which is the individual’s perceived ability to control the symptoms and the degree to which they perceive it can be cured (Leventhal et al., 1980, 2008, 2016). The model further distinguishes between illness-relevant prototypes and representations. Prototypes are relatively fixed “memory structures” that are available to an individual to construct situation-specific representations. For example, an individual’s prototype of themselves and their experiences when healthy or ill is drawn on when constructing a representation of acute and chronic conditions.

Leventhal’s CSM (hereafter referred to as ‘the model’) posits that self-management depends on illness beliefs and that a change in these beliefs can encourage more effective self-management (McAndrew et al., 2010). Improved health outcomes have been reported with CSM-based behaviour-change interventions in rheumatoid arthritis (Hoyt and Stanton, 2012), asthma and diabetes (McAndrew et al., 2008) and other chronic conditions (Broadbent et al., 2009; Siemonsma et al., 2013). While there is an abundance of research exploring individuals’ illness representations, this is yet to be explored in RP, despite the poor management, quality of life and understanding of this difficult condition. The aim of this study was to address this knowledge gap by exploring illness perceptions of individuals with RP, in order to provide a theoretical basis for future intervention development. Two objectives were developed: (1) improve understanding of the illness perceptions of individuals with primary RP; (2) better understand factors associated with adherence and self-management. A qualitative approach was chosen to address these objectives as it allows for an in-depth exploration that centres the diversity of people’s experiences rather than relying on homogenizing a priori assumptions about these experiences (Braun and Clarke, 2013). Semi-structured interviews, the method of choice for our study, allow for gaining insights into participants’ experiences while avoiding inadvertently priming responses, as may be the case in questionnaires (Ogden, 2005). Their open-endedness allows for participants to direct the conversation towards experiences that are meaningful to them rather than those initially considered meaningful by the researcher (Kallio et al., 2016). Qualitative approaches to health psychology thus contribute to a deeper understanding of the subjectivity of illness experiences. These insights are valuable for health psychology practice as they can shed light on the beliefs held by patients and thereby inform interventions that work with these beliefs, e.g., to foster treatment adherence (Leventhal et al., 1992).

## Methods

### Design

A qualitative approach and reflexive thematic analysis (Braun and Clarke, 2006, 2013) were used to explore individuals’ experiences of RP. One-to-one semi-structured interviews were conducted online using Microsoft Teams.

### Participants

A total of 19 participants were recruited, in line with recommendations by Clarke and Braun (2013) and other similar qualitative research in rheumatoid arthritis (Poh et al., 2017; Tehan et al., 2019). Of those, 95% identified as female (*n* = 18) and one participant as male. Thirteen participants were diagnosed with secondary RP and six with primary RP. We included adults aged 18 and over who have had a confirmed diagnosis of Raynaud’s (either primary or secondary) for a minimum of 6 months to take into account adjustment to diagnosis and adherence/non-adherence to treatment. Individuals were required to be fluent in English to participate in the study.

Participants were recruited through social media advertisements posted on the Scleroderma and Raynaud’s UK charity (SRUK) social media (Facebook and Twitter) pages in June 2020, inviting people with Raynaud’s to participate. The advertisements included a link directing participants to a survey to register their interest and provide their email address or telephone number. Participants were based in the UK, but we did not collect further data on their locations.

### Ethical considerations

Ethical approval was obtained from the University of Bath Psychology Ethics Committee in June 2020 (ref: 20-114) and this study was conducted in accordance with the recommendations for research by the British Psychological Society (British Psychological Society, 2018), such as respecting participants’ privacy and confidentiality, and their ability to consent. All participants provided consent via email and verbal consent prior to interviewing, and they were informed that the data was strictly confidential and would be anonymised. Interview audio recordings were stored on a secure drive with access restricted to the researchers. All audio recordings were transcribed and deleted within 4 weeks of the respective interview taking place. Names and other identifiable information were removed from the transcript at time of transcription. Names provided in the Results are pseudonyms.

### Procedure

Interviews were conducted by the first author (MS) in July 2020, over the video-communications platform Microsoft Teams which provides secure encryption and enables audio recording (Ilag, 2018). Data collected using video interviews has been found to be of similar quality to face-to-face interviews, with video interviews having the added benefit of removing geographical barriers and providing a safe space of one’s own choice (Lo Iacono et al., 2016). Interviews were held for 22 to 47 min, with an average duration of 36 min. Orthographic transcription was adopted; the recordings were written word-for-word verbatim, including pauses, fillers, and nonverbal sounds (Clarke and Braun, 2013).

### Interview schedule

The interview schedule was developed around the identified gaps in the literature on Raynaud’s (Daniels et al., 2018; Pauling et al., 2018) to address the study aim. See Table 1 for a summary of the topic guides and their purpose, with examples of prompts.

**Table 1.** Overview of the topic guides, prompts and their purpose.

Topic guide	Examples of prompts	Purpose
1. Warm-up	What triggers your Raynaud’s?	Ease the participant into the conversation
2. Illness representations (see Leventhal et al., 2016)	Can you tell me about the symptoms you experience?	To understand perceived symptoms, causes and consequences of Raynaud’s
3. Knowledge and information (see Daniels et al., 2018)	Can you tell me about your understanding of Raynaud’s?	Sources of information and level of understanding
4. Adherence to advice (see Daniels et al., 2018)	Can you tell me about how well you follow the advice given to you?	Explore participant’s compliance with advice and what works
5. Important outcomes for people with Raynaud’s (see Leventhal et al., 2016)	Can you tell me about your main concern with Raynaud’s?	To understand important areas of focus for participants

### Data analysis

Reflexive thematic analysis (Braun and Clarke, 2006, 2013) with a mixed deductive-inductive approach and critical realist epistemology was used to analyse the interviews. The CSM (Leventhal et al., 2016) was used as a theoretical lens to guide the analysis. Its five dimensions of identity, cause, timeline, consequence, and controllability served as the framework for understanding illness representations, addressing the first research objective of better understanding affected people’s perceptions of Raynaud’s. The second objective of exploring factors associated with adherence and self-management was not mapped deductively. Braun and Clarke’s (2006) six-stage guidelines for reflexive thematic analysis were followed to identify, analyse, and report themes from within the data. See Table 2 for a fuller description of the analysis process. To ensure analytic rigour, the transcripts, codes, and themes were discussed between [author initials] as part of the dissertation supervision process. The participant quotes used to illustrate the themes were selected for clearly speaking to different aspects of the themes and with an intention to represent as broad a range of participants as possible.

**Table 2.** Stages of data analysis.

Stage	Description
1	Familiarisation began during interviews, initially forming preliminary analytical thoughts and continued during the interpretative process of verbatim transcription conducted manually, allowing for deeper immersion into the data. Throughout active re-reading of the dataset, interesting extracts were highlighted, and initial ideas reflectively noted
2	Initial codes were produced and organised into meaningful groups. The coding process was reflective, iterative, and bidirectional; codes were adapted as familiarisation of the data increased, and after all the transcripts were coded, this process was repeated to redefine or discard codes irrelevant to the research aim. Given the inductive and deductive approach to analysis, codes were interpreted semantically and latently, addressing both the surface meaning conversed by the participants and the researcher’s deeper interpretation of this meaning through a theoretical lens
3	After coding the dataset, all codes were listed and actively analysed for shared concepts, to develop subthemes and themes
4	The themes and subthemes were observed using Patton’s (1990) dual criteria to assess internal and external homogeneity; changes were made accordingly. Following careful reiteration of stages 2 and 3 of the analysis, codes were further refined as new insights and patterns of meaning were identified, and themes were redefined
5	Theme names were finalised and explicitly defined in a narrative, in relation to multiple extracts identified from the dataset
6	The analytic report was synthesised

### Reflexive statement

This research was primarily conducted by me, [Author 1], as part of my master’s dissertation on a health psychology programme. I have prior experience engaging with healthcare professionals and personal familiarity with managing chronic health conditions, which shaped my awareness of patient-healthcare dynamics. While conducting interviews with individuals with Raynaud’s, I remained mindful of my own assumptions and engaged in self-reflection to ensure that participants’ voices guided the findings. To enhance trustworthiness, I sought feedback from supervisors, maintaining transparency in my analysis. While complete objectivity is unattainable, I have strived to present an authentic and ethical representation of participants’ experiences.

## Results

Three themes and nine subthemes were generated from the data (see Table 3), capturing the experiences and representations of individuals with RP: 1) illness representations, mapped deductively onto the five dimensions of the CSM (Leventhal, 2016), 2) coping responses; 3) advice and guidance.

**Table 3.** Summary of themes and subthemes.

Themes	Subthemes
Illness representations	Identity beliefs, cause attribution, timeline, consequences, controllability
Coping response	Avoidance coping, approach-oriented coping
Advice and guidance	Role of healthcare professionals, support groups

### Illness representations

The participant’s illness perceptions and experiences of Raynaud’s are captured within this theme and have been deductively mapped onto the five dimensions of the CSM.

#### Identity beliefs

Individuals often identified their Raynaud’s in terms of physical sensations, described in terms of pain, numbness, and cold extremities, and often associated with colour changes: “*Um, if it is really bad it does get a bit painful, um so like after my hands go white, I have to warm them up and that can be really intense like pins and needles*” (James).

Participants felt confident in identifying episodes of Raynaud’s; perhaps due to the specific symptoms experienced, or the familiarity of living with a chronic illness. Self-awareness was often demonstrated through cautious self-monitoring to avoid symptoms and situations that could trigger an episode. This self-awareness enabled participants to instinctively identify their symptoms as Raynaud’s: “*I just feel my hands go cold, and that’s how I identify now and it’s just instinct, that it’s an attack rather than just being cold*” (Heather).

Although these physical sensations were predominantly experienced as ‘symptoms’, participants mostly identify their Raynaud’s in terms of the impact on mobility: “*I can handle the Raynaud’s I can handle the blood circulation cutting off but it’s the pain for me is the worst, which affects me the most, that’s what limits me*” (Rachel). The impact on mobility was attributed to the behavioural experiences of individuals; experiences that were observable by others. When an episode occurs, it is difficult to make fine motor movements, causing functional limitations. These limitations are associated with the pain and numbness experienced as the hands are either too painful to function or the loss of feeling makes it challenging to complete daily tasks: “*often I find it quite difficult to use my hands; so anything that particularly requires a kind of um like fine motor movements, so things like um you know if I’m in a shop and um it’s flared up and I have to pay using a bank card, often I can’t get my bank card out of my purse*” (Amanda). Participants describing that they “*just deal with it*” (Michelle) indicates that these daily limitations are accepted as part of living with a chronic illness. While this acceptance suggests an integration of Raynaud’s with participants’ sense of self, the condition was nonetheless associated with interruptions to their usual activities. These disruptions were in turn positioned as having negative impacts on one’s sense of self; “*You feel a bit of a fool sometimes*” (Sharon). The description of Raynaud’s in terms of debilitating symptoms, points to the development of an illness identity that is limiting and ‘disabling’.

#### Cause attribution

Despite an understanding of the pathogenesis of Raynaud’s, its cause was mostly attributed to triggers and exacerbating factors of Raynaud’s, such as stress, cold and lifestyle factors. Causes were often associated with factors amenable to self-management, such as diet and exercise. This motivated participants to modify their health behaviours, perhaps because of their involvement in the perceived cause, and their inability to control environmental factors, such as cold climates. Participants’ illness representations were for example monitored and appraised depending on the success or failure of their coping efforts of healthy eating: “*I’ve been on quite a strict diet, been following keto paleo diet and cut out an awful lot of things I used to have you know, alcohol, coffee, different things and I feel an awful lot better*” (Sandra). Yet, some participants had difficulty identifying any cause, reporting an unpredictable nature of the episodes, “*but sometimes it happens on its own or seems to just happen on its own*” (Heather). Uncertainty around the causes of Raynaud’s made it difficult to prepare for an episode, providing a sense of low control over the symptoms.

#### Timeline

Raynaud’s was generally understood to be a long-lasting, chronic illness. This was especially evident amongst those who were more accepting and felt in control of their illness: “*It’s not great but you just kind of like have to assume that it is a chronic disease and it’s not going to go anywhere, so it is up to you to have a better um quality of life by not getting so many attacks*” (Julie). All participants were concerned about the future, with uncertainty around disease progression, worsening of symptoms and availability of treatment. “*It’s the condition getting any worse I think and it affecting my life really, because at the moment it’s manageable. But in the future who knows, you know who knows what’s going to happen*” (Deborah).

A clear difference existed between types of Raynaud’s and the concerns around disease progression. Those with primary Raynaud’s were mostly concerned about the potential underlying conditions and the uncertainty of “*whether it’s going to continue to get worse or not*” (Amanda), as predictors of progression to secondary Raynaud’s are not fully understood. Worries among those with secondary Raynaud’s centred on the loss of hand function and symptoms worsening.

#### Consequences

The reported consequences of Raynaud’s were solely negative. Across participants, episodes were reported as regular and associated with a substantial detrimental impact on quality of life. For some, these consequences were mild and manageable, described as “*annoying*” (Amanda), but others were impacted more severely, such as limited hand functioning interfering with routine activities. This removed a sense of control over the illness, and participants felt withdrawn from certain situations: “*I’ve got children and they could be having you know families… I haven’t been able to go out in the winter and play snowballs with them and build a snowman. I’ve missed out on that because I couldn’t do it because my hands hurt*” (Deborah).

Equally, the inability to carry out seemingly small but valued tasks, such as wearing jewellery and ‘heels’, negatively impacted self-identity. Participants’ curtailed ability to express themselves was accompanied by a loss of identity and feelings of powerlessness and distress: “*when my hands are very bad, I struggle to get dressed. I struggle now to do buttons, tie shoelaces, um I can’t wear necklaces anymore I can’t do any fasteners or anything like that… which some of this might not sound like a big deal but it’s all part of who you are. I like to make an effort you know and when my hands are really bad, you can’t do these things, you can’t, and so it changes who you are … when it stops me from doing stuff I like, then I feel like it just ruins my life*” (Linda).

When participants relied on their hands for their career, both symptoms and the uncertainty of disease progression resulted in low mood: “*I’m an artist and graphic designer so you know my hands are very important to me, so um emotionally I was very frightened and because I’m worried about my career … I’d sort of have to not use that and maybe hide it [hands] a little bit from everyone else as well, um because I didn’t sort of want, I don’t know my boss or whatever to think there was something wrong with me and I couldn’t do my job*” (Kelly). This hypervigilance, exemplified by Kelly hiding her hands from her manager, indicates anxiety and vulnerability to a self-maintaining vicious cycle of stressors and symptoms. Perceiving consequences as severe led participants to constantly prepare for episodes of Raynaud’s, negatively impacting their quality of life.

#### Controllability

Raynaud’s was perceived as an incurable but controllable disease by most participants. Treatment preferences revolved around taking a holistic approach and using self-management strategies to prevent symptoms by “*planning ahead*” (Janet). Participants emphasized their self-efficacy and, in some cases, took complete accountability for their symptoms, believing that it resulted from being unprepared: “*I feel like I’m in control of my condition, um and generally if I get an attack then I can say it’s my own fault, it’s very unusual but it comes out of the blue when I’m not expecting it*” (Theresa).

Although some participants felt that “*my Raynaud’s is still really well and under control*” (Heather), others had low perceived control. Behaviours were engaged in to reduce the frequency and severity of the episodes and self-management strategies mainly consisted of wearing gloves and keeping the body warm, and although this was not effective for everyone, most participants relied on these. “*I mean I try and keep myself warm when I go outside so keeping my core warm; you’ll always see me with a pair of gloves … I try to prevent the attacks as much as I can by keeping myself warm*” (Rachel).

Focusing on “*a healthy diet*” (Julie) and exercise was another way that participants attempted to control their symptoms. This corresponds with the participants perceived causal attributions of their Raynaud’s to controllable, lifestyle factors such as stress, diet, and exercise: “*so my self-management is exercise; I go running, I walk pretty much every day with my job anyway, anything like two to four miles with work*” (Sarah).

Some participants faced difficulty gaining control as it was impractical to self-manage by always keeping warm. This difficulty was often accompanied with feeling isolated as others did not understand their illness and would simply suggest keeping warm. It was notable that the only male participant expressed a lack of control over the Raynaud’s, opting for immediate relief of symptoms as opposed to prevention: “*a lot of people will say I’ve got cold hands too, man up or something … it’s not debilitating my life to the point that I can’t function … I’ll wear gloves and I’ll take like 2 pairs of gloves but I’m not going to act in like a preventative way … I’m not going to go to the doctor for that you know*” (James).

Mixed views existed around the use of medication to treat Raynaud’s. Many participants avoided medication and opted for self-management, while for others, medication was a vital aspect of managing Raynaud’s and the impact it had was highly praised as “*the difference is incredible just on a really low dose, really good*” (Sarah). These participants gained a sense of control by using pharmacological treatments to manage their illness, although the degree to which they were involved in controlling their illness was low. Preconceived beliefs of Raynaud’s existed that self-management alone was not sufficient for treatment, and they often relied on medication. The pharmacological side-effects were also discussed, underlining feelings of ambivalence and encouraging participants to avoid medication: “*I used to take Sildenafil, but I had a headache all the time because I took it three times a day and you know it was too debilitating really for that to happen every time I’ve taken it*” (Linda).

### Coping responses

Two coping responses were identified: avoidance coping, referring to maladaptive coping strategies such as denial and unhelpful behavioural responses, and approach-oriented coping, referring to adaptive coping strategies, such as problem-solving behaviours, acceptance and information seeking.

#### Avoidance coping

Avoidant behaviour was reported as the main coping mechanism, primarily consisting of withdrawing from situations that exacerbate symptoms. An example is the reluctancy to go outside and withdrawing from social situations to avoid symptoms: “*I stay indoors a bit more probably than I normally would. I’m reluctant to go outside, um heating goes on and I have to turn the heating up*” (Deborah). Another participant reported seeking immediate relief for their symptoms by running their hands under a hot tap despite awareness of the long-term damage this can cause: “*my rheumatologist told me off for doing it but I will run my hands under a hot tap to bring it back because I sort of need that sharp you know*” (Rachel).

#### Approach-oriented coping

Responding to the demands of chronic illness by using problem-focused coping involved attempts to manage symptoms, control pain, and seeking information, including planning ahead and prevention: “*yeah just trying to keep active, eating healthy … so if I know that I’m going to the aisle in a supermarket then just going with an extra cardigan or jacket or whatever, or if I know that it’s windy and it’s cold then I just make sure that I always have a pair of gloves in my handbag*” (Julie). Another participant focused on stress-reduction techniques, an adaptive coping style that targets the cause of the problem: “*Um I know if it’s stress related then I try and do breathing exercises and do a bit of yoga*” (Pamela).

Participants also reported habitual self-management routines that enabled them to remember their gloves and remain disciplined. The importance of wearing gloves and protecting their hands was expressed, and methods such as placing the gloves by the door ensured they were not forgotten: “*it’s mostly just staying warm really and having creative ways of making sure that your core is warm, your gloves are available; you don’t get the opportunity to forget your gloves you just make sure that um your gloves are warm and that you walk past them on the way out of the house and you don’t forget them, that’s probably the biggest thing*” (Theresa).

Some participants emphasised creating routines and maintaining positive health habits. Given the unpredictable episodic nature of Raynaud’s, it may be difficult to maintain a routine. Nonetheless, there was a sense of observable positive differences in their health when maintaining a routine: “*I do notice that when I’m more disciplined and like if I eat better and um I feel better and in general like if I have a bad week or like I’ve been drinking more than I should or I’ve been eating pain au chocolates and croissants [laughs] and things like that, I do notice that these affect my health*” (Julie).

### Advice and guidance

Illness representations are defined by perceptions of the self which are formulated from previous experiences, such as social interactions with friends, family, and healthcare professionals (Benyamini and Karademas, 2019). Advice and guidance depict the interactions with healthcare professionals and the function of support groups for emotional and practical support.

#### Role of healthcare professionals

Participants reported a lack of awareness and understanding of Raynaud’s, especially amongst general practitioners (GPs). Some GPs tended to dismiss individuals, were unaware of Raynaud’s and lacked general understanding of treatment options. This resulted in delayed diagnoses, inadequate or no advice and guidance for self-management, and a delay in treatment; all of which negatively impacted participant’s adjustment to the illness: “*I only got the formal diagnosis about 9 months later when I moved GPs and they got the notes and they went through it with me and they said, ‘Oh and of course you have Raynaud’s’, and I was like ‘Do I?’ [laughs]… it was never regarded as a major thing which is unfortunate because actually, I think had I had a bit more advice quite early on, there would have been times I would have been more confident about things*” (Janet).

Other participants did have a positive GP experience. There was however an acknowledgement that poor understanding existed amongst healthcare professional and when participants had positive experiences, they expressed themselves as fortunate to have a GP with an understanding of Raynaud’s. Advice given was mostly related to retaining warmth and sometimes consisted of advice to minimise stress: “*just try and avoid stressful situations*” (Pamela). Participants expressed that advice to minimise stress lacked practical support and they were dismissed by the GP with little guidance for self-management: “*I think it would be good for a GP to talk through with me where my symptoms put me on things and how careful I really need to be and if how often it is triggering would be damaging and would lead to long term damage*” (Janet).

As secondary Raynaud’s is usually more severe than primary, it was related to receiving better quality advice from specialists, whereas those with primary Raynaud’s were generally dismissed and were “*just told it was an annoyance*” (Sharon). This was associated with emotional consequences for participants; in an attempt to seek information for their illness, they were left frightened and unsupported, which may explain the differences in adherence to self-management behaviours amongst participants: “*My GP eventually knew about Raynaud’s, but he just left it there, ‘You have primary Raynaud’s’ that’s it’*” (Julie).

#### Support groups

The majority of participants talked about relying on the experiences of others with Raynaud’s for support and guidance. The use of support groups on online forums were described as “*an absolute godsend*” (Kelly). The unique experience of living with Raynaud’s was reportedly only understood by others with the same illness, “*only someone else with Raynaud’s would understand*” (Sarah). These online interactions were also beneficial to coping with the illness as it provided an emotional outlet, emphasising the importance of shared experiences: “*you go on there and ask a question and you get so many responses and people who are also going through the same so you know it is part of the condition; so yeah and it’s just good to know and speak with other people with it as well so um yeah I find it really useful, really, really handy*” (Rachel). These shared experiences allowed individuals to adopt new strategies to assist their self-management as well as share their own experiences: “*Well hints and tips are really good so sharing hints and tips and things because there are silly little things that can help*” (Heather).

Whilst many participants found these shared experiences invaluable, there were also negative perceptions. These could come out of a mismatch of expectation and experience, where the participant’s expectation was to gain advice and receive answers, but their experience was primarily of members seeking sympathy. A similar negative emotional connotation was echoed when support groups were associated with a reminder of being ill. This participant describes how observing others’ symptoms worse than their own through support groups led to feelings of uncertainty around disease progression, perhaps as they were not ready to accept the potential consequences of their illness and their low perceived control: “*I did join some of those at one point, to learn a bit more but it started getting me down a little bit. Um because there are a lot of people out there who are a lot worse than me and I kind of wasn’t ready to hear about all of that*” (Deborah).

## Discussion

This was the first study to explore individual’s experiences and illness perceptions of Raynaud’s to better understand adherence, self-management and factors associated with outcome. The CSM appears to provide a useful framework for understanding experiences of Raynaud’s, as the participants’ experiences are represented with the five dimensions of the CSM. Three interrelated themes were identified: ‘*illness representations’*, ‘*coping response’* and *‘advice and guidance’*.

Participants’ illness representations were shaped by the impact of symptoms on daily activities, which were consistently described as negative. Causal attribution for the symptoms tended to be attributed to factors amenable to self-management (stress, cold, and lifestyle) rather than underlying pathogenesis, but perceptions about the controllability of the illness varied. Consequences included disruptions to routine activities and feelings of powerlessness and distress, with thoughts about the future marked by uncertainty about disease progression.

Participants’ descriptions of Raynaud’s in terms of the constraints it placed on their everyday lives strongly suggests that identity beliefs were more closely related to the impact of the disease than its severity. Our findings point to negative identity beliefs that may result in focus on functional limitations which may in turn further restrict individuals’ lives through avoidant behaviour (Brooks et al., 2013). This focus on functional limitations provides an important target for treatment, particularly if those with RP feel ‘engulfed’ by the condition.

The perceived ‘causes’ of Raynaud’s (episodes, rather than underlying cause) were mostly attributed to controllable factors. Echoing previous findings, the most salient reported causes were stress and exposure to cold temperature (Pauling et al., 2018), which are arguably fairly difficult to control as otherwise the distress and anxiety expressed about the consequences would not be present. This is consistent with attribution theory (Weiner, 1986), which suggests that participants’ causal attributions of their illness are directly related to behavioural performances, such as coping responses and self-management. In our case, individuals who attribute the cause of Raynaud’s to lifestyle factors such as diet, may focus more on integrating a healthy diet as a way of managing their illness (Weiner, 1986), despite there being a lack of robust evidence to support these notions – this may reflect a lack of education on part of the patient and perhaps also the GP, as reported by participants. Intervention may thus first seek to clarify and educate patients on the causal factors amenable to self-management and the evidence for existing treatments (Daniels et al., 2018).

Raynaud’s was perceived as a chronic and potentially worsening condition, with anxiety and low mood around uncertainty of disease progression. This is contrary to research which suggests only a small proportion of those with primary Raynaud’s go on to develop serious conditions that belie secondary Raynaud’s (Daniels et al., 2018). These concerns were strongly related to poor health-related quality of life and poor self-management, perhaps due to low perceived control; findings that were echoed in recent research in systemic sclerosis (Brijs et al., 2019; Frantz et al., 2016) and a recent paper which indicated that anxiety contributed to both symptom severity and quality of life (Broughton et al., 2024). The perceived uncertainty of the illness, not only around progression in the longer term but also about predicting the onset of episodes, further led to hyper-vigilant self-monitoring, behaviours also reported in a focus group study on patients with secondary Raynaud’s (Pauling et al., 2018). Treatments should therefore seek to promote an approach to self-management that both acknowledges its value for managing the condition but discourages hyper-monitoring. Worries about disease progression differing between primary and secondary RP (progression to secondary RP and loss of hand function/worsening of symptoms respectively) emphasize the need for a tailored approach that is responsive to differences between types of the illness (Broughton et al., 2024).

Consequences of Raynaud’s were perceived to be solely negative, affecting every aspect of participants’ lives, including their ability to work and socialise, albeit during Raynaud’s episodes. Some participants appeared to be poorly adjusted to Raynaud’s, focusing on a loss of identity and abilities, which is purported to be derived from attempting to maintain previous representations of the self (Aujoulat et al., 2007), suggesting the illness is rejected from their identity. Shifting one’s identity to adapt is an important ongoing process of adjustment to chronic illness (Helgeson and Zajdel, 2017), but when the illness stands in conflict with valued activities this process becomes difficult and can lead to distress (Stanton et al., 2007). Rejection of a chronic illness from one’s identity is often associated with more symptoms and poor self-management (Oris et al., 2016), emphasising the importance of understanding illness representations and their integration with individuals’ identities to target interventions. This consideration of identities also relates to gender; although this study was limited to one male participant, it is notable that his account echoed a sense of self-reliance and self-confidence previously described among men with chronic illness (Smith et al., 2009). His statement that he would not seek help nor act preventatively suggests that gendered expectations around one’s relationship to the illness may contribute to the uptake of self-management strategies. However, we are cautious to generalise from one participant. Further studies may wish to examine the gendered experiences of Raynaud’s in closer detail.

Raynaud’s was mostly perceived as controllable, but with some uncertainty on how to control (rather than prevent) symptoms. Consistent with previous research, mixed efficacy was found with pharmacological treatments (Hughes et al., 2015), concerns existed around side-effects of such treatments (Pauling et al., 2018) and the efficacy for non-pharmacological treatments could neither be confirmed nor refuted (Daniels et al., 2018). Our findings suggest that medication was perceived by some as a threat to their sense of control that was gained through self-management, while others felt increasing control through medication. This is one area where individual difference do prevail; while many struggle with older types of medication, with time and titration, positive benefits can be seen and there is by no means a one size fits all approach to current treatments. Overall, a preference was observed for controlling Raynaud’s using self-management strategies that are in line with recommendations by NICE guidelines (NICE, 2020), such as wearing gloves, keeping warm, exercising, and attempting to reduce stress. These are very much ‘common sense’ approaches rather than empirically grounded recommended interventions, and as reflected in our sample, are not consistently effective at preventing or alleviating symptoms. This may be due to inconsistent implementation of these strategies, or an oversimplification of what interventions might be successful in managing and treating Raynaud’s episodes, or both. Participants’ reluctance towards medication, the absence of efficacious behaviour-change interventions and the emerging body of work on psychological factors in Raynaud’s all taken together presents a clear argument for more development in this field. In other chronic health conditions, randomised controlled trials have shown statistically significant improvements in health-related outcomes from an intervention targeting cognitive treatment of illness perceptions (Siemonsma et al., 2013) and represent a compelling direction for future treatment for Raynaud’s (Cameron and Hagger, 2025). The relevance of the CSM framework in Raynaud’s is clear both here and elsewhere (Broughton et al., 2024). The Revised Illness Perception Questionnaire (IPQ-R; Moss-Morris et al., 2002) should be considered for use in clinical assessment of those with Raynauds, used to identify key illness cognitions that can form the basis of individualised formulation and effective behaviour change interventions (Wearden and Peters, 2008). Such interventions might include goal setting, self-monitoring, education/information, environmental modifications, stress management cognitive behavioural techniques (Michie et al., 2014), all of which would align to NICE recommendations our research findings and the existing evidence base on key psychological factors in Raynaud’s. Indeed, interventions have been found to be effective in targeting anxiety, pain, and adherence through stress management and coping skills training in rheumatological interventions (Van Middendorp and Evers, 2016).

The illness representations were interrelated with coping responses. Perceiving more serious consequences and low perceived control suggested avoidance coping as participants did not feel equipped to manage symptoms. They therefore withdrew from situations that could exacerbate symptoms, as a way of gaining control. Previous research has suggested that maladaptive coping is associated with perceiving more illness consequences, resulting in poorer quality of life (Hallas et al., 2011; Willemse et al., 2019). Maladaptive behaviour may lead to short-term advantages for the participants, such as a reduction in symptoms, stress and self-consciousness. These advantages may be helpful at an acute level, but avoidant behaviour often acts as a negative reinforcer which can result in repeating an unhelpful coping response (Leventhal et al., 2016) and may lead to negative long-term consequences (Roesch et al., 2005). These findings reinforce the need to target illness beliefs for better health outcomes and engaging in more adaptive coping strategies, as previously reported in self-management interventions guided by the CSM (Karekla et al., 2019).

Conversely, a desire to manage symptoms, gain new skills, and seek information indicated better adjustment to Raynaud’s through approach-oriented coping, consistent with findings by Keefe and colleagues (2002). An adaptive coping style that targets the cause of the problem and may result in alleviating symptoms, improving quality of life and health outcomes (Merkes, 2010). Some participants emphasised creating routines and maintaining positive health habits, all of which are key predictors of adaptive coping outcomes (McSorley et al., 2014). The development of prompts and cues reported by participant is grouped in the ‘associations’ category of the behaviour-change taxonomy, which is often utilised in developing behaviour-change interventions (Michie et al., 2013). The use of cues may encourage a shift from self-management behaviours that are conscious and deliberate, to actions that are automatic (Leventhal et al., 2016) and may be leveraged in interventions for Raynaud’s self-management.

Advice and guidance reflect the interactions with healthcare professionals and the function of support groups for emotional and practical support, albeit not always successfully satisfying the need. Most participants reported an unacceptable lack of awareness and understanding of Raynaud’s amongst GPs, resulting in delayed diagnoses, inadequate or no advice for self-management and delay in treatment, therefore, impacting their adjustment and coping with illness. Little guidance for self-management was provided, especially for those with primary Raynaud’s who are not referred to specialists. Support groups enabled people to share similar experiences, reduce feelings of being alone with the illness and find a community. As found elsewhere (Ashtari and Taylor, 2023) experiences were not all positive, but could be frustrating and had the potential to increase rather than decrease distress. Where it was deemed to be supportive, learning strategies to self-manage and peer support were considered the most useful aspects. The advice and guidance received can inform prototypes of participants’ illness representations and subsequently their coping style and adherence to self-management, so engagement with support groups should be encouraged but with caution; the potential for negative experiences, social comparison and competitiveness around illness identity (also noted by Embuldeniya et al., 2013) should be taken into account. It is also the case that some may not have access to online support groups for reasons relating to health inequalities; this risks isolation for those who may benefit from the positive aspects online support groups bring. From this perspective, moderated, health professional led support groups would offer a potentially safer more accessible option, but this again is also subject to potential obstacles.

These findings suggest that targeting illness cognitions and adherence to NICE recommended self-management strategies may improve health-related outcomes for people with RP, as recommended by Daniels and colleagues (2018). However, development of interventions tailored to RP are essential. Given the individual differences in self-management of RP, a stepped care approach may be beneficial. In the UK, this is recommended by NICE for other long-term conditions (National Collaborating Centre for Mental Health, 2018) and in line with the NHS long-term plan outline for the integration of mental and physical healthcare for people with anxiety and low mood in the context of long-term conditions (NHS, 2019).

### Limitations

While this study has made a novel contribution to the literature, it is not without limitations. Due to the recruitment of self-selected participants via RP organisations, our participants may already be actively seeking information for their RP. Future research may wish to explore illness perceptions in participants who do not seek support via RP organisations. Differences in experiences existed between primary and secondary RP. Although this study did not set out to distinguish by types of RP, future research may benefit from exploring these separately for more in-depth analyses. Furthermore, the findings should be considered in the light of most participants being female and of the study being focussed on the UK context. Finally, given the impact of cold climate on RP (Pauling et al., 2018), it is important to acknowledge that this study was conducted in the summer, potentially affecting the recollections of participants episodes triggered by more pronounced cold exposure. The study was however conducted in the UK, which experiences temperate variation even over summer months. Future research interested in the relationship between illness perceptions and factors related to health outcomes across different populations should consider measuring illness representations for RP using the Revised Illness Perception Questionnaire (IPQ-R; Moss-Morris et al., 2002), which is the most accepted and commonly used measure for the assessment of illness cognitions across populations (Järemo et al., 2017). Future studies may also want to further explore how individual differences may impact on perceptions of controllability, in particular in the context of living with multimorbidity. Such studies can complement the findings reported here to develop or adapt interventions for individuals with Raynaud’s, who are currently limited in the support that is available to them.

### Conclusion

This was the first study to explore individual’s experiences and illness perceptions of Raynaud’s to better understand adherence, self-management and factors associated with outcome. The CSM provided a useful framework for understanding experiences of Raynaud’s. Illness representations were interrelated with coping responses and experiences with receiving advice and guidance, and while there were broad consistencies, this work highlighted key factors that may be associated with poorer adjustment. Illness cognitions should be considered important treatment targets that may improve quality of life and symptom experience in Raynaud’s phenomenon, however further work is needed.

## Data Availability

The data that support the findings of this study are available from the corresponding author upon reasonable request.*
